# An attention-based effective neural model for drug-drug interactions extraction

**DOI:** 10.1186/s12859-017-1855-x

**Published:** 2017-10-10

**Authors:** Wei Zheng, Hongfei Lin, Ling Luo, Zhehuan Zhao, Zhengguang Li, Yijia Zhang, Zhihao Yang, Jian Wang

**Affiliations:** 1 College of Computer Science and Technology, Dalian University of Technology, Dalian, China; 2 College of Software, Dalian JiaoTong University, Dalian, China

**Keywords:** Attention, Recurrent neural network, Long short-term memory, Drug-drug interactions, Text mining

## Abstract

**Background:**

Drug-drug interactions (DDIs) often bring unexpected side effects. The clinical recognition of DDIs is a crucial issue for both patient safety and healthcare cost control. However, although text-mining-based systems explore various methods to classify DDIs, the classification performance with regard to DDIs in long and complex sentences is still unsatisfactory.

**Methods:**

In this study, we propose an effective model that classifies DDIs from the literature by combining an attention mechanism and a recurrent neural network with long short-term memory (LSTM) units. In our approach, first, a candidate-drug-oriented input attention acting on word-embedding vectors automatically learns which words are more influential for a given drug pair. Next, the inputs merging the position- and POS-embedding vectors are passed to a bidirectional LSTM layer whose outputs at the last time step represent the high-level semantic information of the whole sentence. Finally, a softmax layer performs DDI classification.

**Results:**

Experimental results from the DDIExtraction 2013 corpus show that our system performs the best with respect to detection and classification (84.0% and 77.3%, respectively) compared with other state-of-the-art methods. In particular, for the Medline-2013 dataset with long and complex sentences, our F-score far exceeds those of top-ranking systems by 12.6%.

**Conclusions:**

Our approach effectively improves the performance of DDI classification tasks. Experimental analysis demonstrates that our model performs better with respect to recognizing not only close-range but also long-range patterns among words, especially for long, complex and compound sentences.

## Background

Therapy with multiple drugs is a common phenomenon in most treatment procedures. Drug–drug interactions (DDIs) occur when one administered drug influences the level or activity of another drug. DDIs often lead to unexpected side effects or a variety of adverse drug reactions (ADRs) [1]. DDIs are one of the main reasons for the majority of medical errors. Based on their financial, social and health costs, the recognition and prediction of DDIs, and hence their prevention, can greatly benefit patients and health care systems [2].

Today, huge amounts of the most current and valuable unstructured information relevant to DDIs are hidden in specialized databases and scientific literature. Text mining based on computerized techniques may recognize patterns and discover knowledge from various available biological databases and unstructured texts [3–5]. Hence, the use of text-mining techniques to recognize DDIs from databases and texts is a promising approach. In addition, this technique contributes to the automation of the database curation process, which is currently performed manually, and the building of a biomedical knowledge graph [6].

To improve and evaluate performance with respect to classifying DDIs from biomedical texts, DDIExtraction challenges in 2011 (DDI-2011) [7] and 2013 (DDI-2013) [8] were organized successfully. Each challenge provided a benchmark corpus. DDI-2013 not only focused on the identification of all possible pairs of interacting drugs (the detection task) but also proposed a more fine-grained classification of each true DDI (the classification task). The two tasks are regarded as binary and multi-class classification problems, respectively. In particular, the DDI-2013 corpus [9] consists of texts selected from the DrugBank database (DB-2013 dataset) and MEDLINE abstracts (ML-2013 dataset). They contain sentences with different styles. The DB-2013 dataset contains short and concise sentences, while the ML-2013 dataset usually contains long and subordinated sentences that are characterized by scientific language. Overall, the performance of existing DDI classification systems decreases drastically for long and complex sentences [10], which may be one of the main reasons for the lower F-score obtained on the ML-2013 dataset than that on the DB-2013 dataset.

Traditional studies of DDI tasks mainly use machine-learning methods such as support vector machine (SVM). In general, features such as word-level features, dependency graphs, and parser trees are designed manually by SVM-based systems [11–17], which have performed well during the past decade. Natural language processing (NLP) toolkits such as syntactic and dependency parsers are exploited to parse sentences, which unavoidably brings some unexpected errors, especially for long sentences. At present, feature extraction is still a skill-dependent task that is performed on a trial-and-error basis. In addition, it has limited lexical generalization abilities for unseen words.

By contrast, neural network (NN)-based methods are automatic representation-learning methods with multiple levels of representation, which are obtained by composing simple but non-linear modules that each transform the representation at one level into a representation at a higher, slightly more abstract level [18]. Recently, deep neural network models have shown promising results for many NLP tasks [19, 20]. There are two main neural network architectures: convolutional neural network (CNN) and recurrent neural network (RNN).

CNN with a fixed-size convolution window can capture the contextual information of a word, which is similar to the traditional n-gram feature. For the DDI-2013 tasks, these CNN-based systems [21–23] have performed well. However, the best performance (an F-score of 52.1%) on the ML-2013 dataset is not satisfactory. The semantic meaning of a drug-drug interaction in a sentence may appear in a few words before, in between, or after the candidate drug pair. The ML-2013 dataset has sentences with relatively longer and more complex structures than those of the DB-2013 dataset. Thus, some meaningful contexts that are relevant to a particular DDI are possibly non-consecutive, and there may be longer spans among them. However, the goal of CNN is to generalize local and consecutive contexts. Therefore, CNN is potentially weak, especially for learning long-distance patterns. CNN-based approaches that utilize multiple window sizes, dependency paths and sufficiently stacked CNN layers can solve the difficulty of CNN models in learning long-distance patterns in part. However, stacked CNN layers are generally harder to train than gated RNNs. In addition, they all either require much higher computational costs or face errors caused by a dependency parser.

By contrast, RNN with long short-term memory (LSTM) units, which is a temporal sequence model, adaptively accumulates context information of the whole sentence through memory units. Thereby, RNN is suitable for modelling long sentences without a fixed length because it has the power to learn the global and possibly non-consecutive patterns. Moreover, there are some successful RNN-based applications [17, 24, 25] for relation classification. However, words need to be transmitted one by one along the sequence in an RNN. Therefore, some important contextual information (for example, long-distance dependencies among words) could be lost in the transmission process for long texts [26].

Currently, some systems exploit the attention mechanism to address this issue. Attention-based models have shown great success in many NLP tasks such as machine translation [20, 27], question answering [28, 29] and recognizing textual entailments [30]. In the context of relation classification, the attention mechanism, weighing of text segments (e.g., word or sentence) or some high-level feature representations obtained by learning a scoring function allows a model to pay more attention to the most influential segments of texts for a relationship category. Wang et al. [31] propose a CNN architecture based on two levels of attention for relation classification of general domains. The *joint AB-LSTM* model [32] combines a general pooling attention with LSTM for DDI classification. However, related experiments indicate that the introduction of pooling attention fails to improve the performance of DDI classification tasks.

In this work, with simplicity and effectiveness in mind, we extracted DDIs from biomedical texts using an attention-based neural network model called *Att-BLSTM* that uses RNN with LSTM units. First, a candidate-drug-oriented input attention on the representation layer was designed to automatically learn which words are more influential for a given drug pair. Next, outputs of a bidirectional LSTM at the last time step represent high-level features of sentences. Finally, a softmax classifier conducted DDI classification. Experimental results on the DDIExtraction 2013 corpus indicate that our model yields F-score boosts of up to 2.2% and 5.8% over the current top-ranking systems for DDI detection and classification, respectively, in addition to the best F-score on all interaction types, especially for the Medline-2013 dataset on which our F-score outperforms the existing best result by 12.6%. Our model significantly improves performance with respect to three datasets. Experiments demonstrate that our model, with an attention mechanism and fewer features, can better recognize long-range dependency patterns among words in sentences, especially in long, complex and compound sentences.

## Methods

In this section, we describe the proposed network model for extracting relations of drug–drug interactions from biomedical texts in detail.

### Text preprocessing

#### Basic processing

We first completed several common preprocessing steps on both training and test data. A special tag replaced each digit string that is not a substring of a drug entity. A bracket without either of the candidate drugs was deleted. For the generalization of our approach, all drug mentions were anonymized using drug* (* denotes 0, 1, 2, …). Sentences of the test dataset with only one entity or two entities with the same token were filtered out because of the impossibility of a relation.

#### Following-based anaphora

After the DDIExtraction-2013 shared tasks, the error analysis of Segura-Bedmar et al. [10] indicates that one of the most important factors contributing to false negatives in DrugBank texts is the lack of coreference resolution. Rules in our approach were defined for some sentence patterns, including the phrase ‘*following [cataphora word]*’ with a colon, where the two entities of a candidate drug pair are on either side of the colon. We may also call this the resolution of the following-based cataphora. In the subsequent pattern, [w]* denotes one or more words.

##### **Case 1:**drug1 [w]* following [cataphora word]: [w]* drug2 [w]*.

Replaced with: drug1 [w]* following drug2.

##### **Case 2:**[w]* following [cataphora word] [w]* drug2:[w]* drug1 [w]*.

Replaced with: [w]* following drug1 [w]* drug2.

Nevertheless, these rules do not work for the ML-2013 dataset, which has hardly any sentences with the above cases.

#### Pruned sentences

If there are overlong texts in a sentence, except texts between a candidate drug pair, redundant information will decrease the detection and classification performances. Therefore, we pruned each sentence to the fixed input length. After computing the maximal separation between a pair of candidate drugs, we chose an input width that is *n* greater than the separation. Each input sentence was made of this length by either trimming longer sentences or padding shorter sentences with a special token.

### Network architecture

**Fig. 1:**
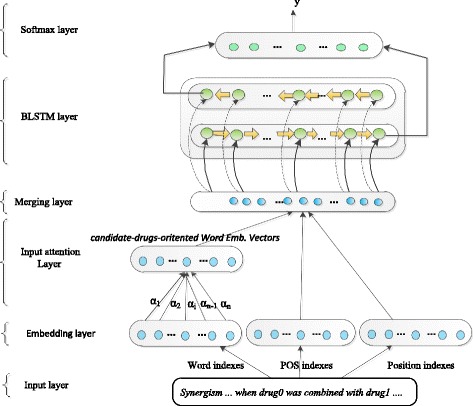
The model architecture with input attention. *Note:* Drug0 and drug1 are the candidate drug pair

Considering the advantages of LSTM in long-distance pattern learning, we still introduced the attention mechanism into our model to overcome the bias defect of LSTM to some extent. Figure 1 gives an overview of the network architecture. The model is composed of six layers: (1) the input layer to accept three types of information, namely, word, part of speech (POS) and relative distances between a word and each candidate drug in an input sentence; (2) the embedding layer to look up tables to encode the above input into real-valued vectors (also called embedding vectors); (3) the input attention layer to weight word-embedding vectors, which are the most influential for the relationship between a pair of special candidate drugs; (4) the merge layer to connect three corresponding embedding vectors into a vector by words; (5) a bidirectional RNN with LSTM units to learn the high-level syntactic meaning of the whole sentence and pass outputs at the last time step to the next layer; and (6) the logistic regression layer with a softmax function to perform DDI classification. The main layers will be described in detail in the following sections.

#### Input representation

After transforming inputs into various embedding vectors, our model feeds them to the subsequent layer. Word embedding, which was proposed by Bengio [33] (also known as distributed word representation), maps words to a low-dimensional and dense real space. It is able to capture some underlying semantic and syntactic information around words by learning from large amounts of unlabelled data. Word embedding reflects the topic similarity among words and improves their generalization to some extent.

#### Input feature

Given a pruned sentence *S* = {*w*_*1*_, *w*_*2*_, …, *w*_*i*_, …, *w*_*n*_}, each word *w*_*i*_ is represented as three features: the word itself, part of speech and position. Each feature group has an embedding vocabulary. The position feature proposed by Zeng [34] was also introduced into our model to reflect the relative distances (*d*_*i1*_ and *d*_*i2*_) between the current word *w*_*i*_ and two candidate drug mentions.

In addition, the semantic meaning of a word *w*_*i*_ that is reflected in a given sentence may be not necessarily consistent with its embedding vector. For example, the word “*effect*” is a noun as well as a verb. However, when it appears in different sentences with different POSs, its embedding vectors are still identical. Therefore, the POS feature is informative for DDI extraction. Our model combined a word with its POS tag (such as NN,VB,DT) to distinguish its semantic meaning in different sentences. We obtained POS tags by using the Stanford Parser [35] to parse above processed sentences.

#### Embedding layer

Each feature group of the input layer has a corresponding embedding layer. Suppose ${{{\boldsymbol{V}}_k}^{l_k}}^{\times {m}_k}$ is the embedding vocabulary for the *k*-th (*k* = 1, 2, 3) feature group, where *m*_*k*_ is the dimensionality (a hyper-parameter) of the embedding vector of a feature and *l*_*k*_ is the number of features in the vocabulary ***V***_*k*_. Each embedding vocabulary can be initialized either by a random process or by some pre-trained word embedding vectors. For a word *w*_*i*_, the embedding layer maps the index token of each feature to a real-valued row vector by looking up its corresponding embedding vocabulary.

#### Input attention

Attention mechanisms have been successfully applied to sequence-to-sequence learning tasks. For relation classification tasks, attention mechanisms are able to learn a weight for each word of a sentence to reflect its level of effect on the final classification result. For a pair of candidate drugs, the DDI tasks aim to classify their relation. It is obvious that not all words contribute equally to the sentence meaning that determines their relationship type. We expected that our model has the ability to determine which words of the sentence are the most influential for the relationship between a pair of special candidate drugs. Therefore, our model applied an attention mechanism to input word embedding for this purpose. We exploited two row vectors ***α*** ^*j*^ (*j*∈1,2), whose size equals the maximum length *n* of the sentence, to quantify the relevance degree of every word *w*_*i*_ of a sentence with respect to the *j*-th drug candidate *e*_*j*_.

1 $$ {\alpha}_i^j= soft\max \left( score\left({\boldsymbol{u}}_{w_i},{\boldsymbol{u}}_{e_j}\right)\right) $$

Here, ${\boldsymbol{u}}_{w_i}$ and ${\boldsymbol{u}}_{e_j}$ are word-embedding vectors of the word *w*_*i*_ and the drug candidate *e*_*j*_, respectively. The *score* function is referred to as a candidate-drug-oriented function, for which we consider the following two alternatives: *dot-score* and cos*-score*:

2 $$ dot\hbox{-} score\left({\boldsymbol{u}}_{w_i},{\boldsymbol{u}}_{e_j}\right)=\frac{dot\left({\boldsymbol{u}}_{w_i},{\boldsymbol{u}}_{e_j}\right)}{m_1}\kern1.75em \left(\ \mathrm{i}\le n\right) $$

3 $$ \cos \hbox{-} score\left({\boldsymbol{u}}_{w_i},{\boldsymbol{u}}_{e_j}\right)=\cos \left({\boldsymbol{u}}_{w_i},{\boldsymbol{u}}_{e_j}\right)\kern2em \left(\ \mathrm{i}\le n\right) $$

Here, the symbols *dot* and cos denote the dot-product and cosine operations on *two vectors* ${\boldsymbol{u}}_{w_i}$ and ${\boldsymbol{u}}_{e_j}$, respectively. *m*_1_ is the dimensionality of the word embedding vector. Then, the candidate-drug-oriented embedding vector ${\boldsymbol{w}}_i^e$ is derived from the combined effects of the two factors ${\alpha}_i^1$ and ${\alpha}_i^2$ acting on the original embedding vector ${\boldsymbol{u}}_{w_i}$ of the word *w*_*i.*_

4 $$ {\alpha}_i=\frac{\alpha_i^1+{\alpha}_i^2}{2} $$

5 $$ {\boldsymbol{w}}_i^e={{\boldsymbol{u}}_{w_i}}^{\ast }{\alpha}_i $$

Here, the symbol * denotes element-wise multiplication. For the sake of comparison, we still provide a non-candidate-drug-oriented function, tanh*-score*:

6 $$ \tanh \hbox{-} score\left({\boldsymbol{S}}_w\right)=\boldsymbol{V}\tanh \left({\boldsymbol{WS}}_w+\boldsymbol{b}\right) $$

where ***V*** and ***W*** are learned matrices, ***S***_*w*_ is the embedding matrix of the sentence, and the size of the function tanh-*score* is same as that of the above ***α*** ^*j*^.

Finally, these vectors, including word embedding ${\boldsymbol{w}}_i^e$, POS embedding ${\boldsymbol{w}}_i^p$ and position embedding ${\boldsymbol{w}}_i^{d1}$, ${\boldsymbol{w}}_i^{d2}$ are concatenated into a new single vector ${\boldsymbol{x}}_i={\boldsymbol{w}}_i^e\mid \mid {\boldsymbol{w}}_i^p\mid \mid {\boldsymbol{w}}_i^{d1}\mid \mid {\boldsymbol{w}}_i^{d2}$ to represent the word *w*_*i*_, where ***x***_***i***_ ∈ *R* ^*m*^ (*m* = *m*_1_ + *m*_2_ + 2*m*_3_). As a result, the sentence *S* is a sequence of real-valued vectors *S*_*emb*_ = {***x***_1_, ***x***_2_, …, ***x***_*i*_, …, ***x***_*n*_}.

#### Recurrent neural network with long short-term memory units

The DDI tasks are relation classifications at the sentence level. In our model, the recurrent layer plays a key role in learning both the long-range and close-range patterns among words in sequence texts.

Theoretically, an RNN [36] has the ability to process a sequence of arbitrary length by recursively applying a transition function to the internal hidden state vector of its memory unit. However, if a sequence is overlong, gradient vectors of the back-propagation algorithm tend to grow or decay exponentially [37–39] in the process of training*.* The LSTM network [39] was proposed by Hochreiter and Schmidhuber to specifically address this issue. LSTM introduces a separate memory cell with an adaptive gating mechanism, which determines the degree to which LSTM units maintain their previous states, and updates and exposes extracted features of the current data input. In our model, we adopted the implementation used by Graves [40].

In addition, RNN is a biased model, where later inputs are more dominant than earlier inputs if it is used to encode a sentence. For the DDI tasks, the effective features for the relation between two candidate drugs might not necessarily appear in front of the current word, and the future words may play a part in the training process of DDI classification. Therefore, our model applied a bidirectional LSTM (BLSTM). For the word *w*_*i*_, the two LSTMs pick up available contextual information along the sentence forwards and backwards, which takes advantage of the previous and future context of the current term to some extent. Thus, BLSTM better captures the global semantic representation of an input sentence. Outputs ($\overrightarrow{{\boldsymbol{h}}_n}$ and $\overleftarrow{{\boldsymbol{h}}_n}$) of the two LSTMs at the last time step *n* are concatenated into a vector ${\boldsymbol{h}}_n=\overrightarrow{{\boldsymbol{h}}_n}\mid \mid \overleftarrow{{\boldsymbol{h}}_n}$ that reflects high-level features of the whole sentence.

### Training and classification

The softmax layer, a logistic regression classifier with a softmax function, classifies the relation between a pair of drugs. It takes the output ***h***_*n*_ of BLSTM as its input. Its output is the probability distribution over each label type for the relation between the candidate drugs in the sentence *S*. The label with the maximum probability value is the interaction type of a candidate drug pair.

7 $$ p\left(y=j|S\right)= soft\max \left({\boldsymbol{h}}_n{\boldsymbol{W}}_S+{\boldsymbol{b}}_S\right) $$

8 $$ \hat{y}=\arg \underset{y\in C}{\max}\left(p\left(y=j|S\right)\right) $$

where the symbol *C* is the set of DDI labels, ***W***_*S*_ is a learned matrix that maps the vector ***h***_*n*_ linearly to the number of DDI labels, and ***b***_*S*_ is a learned bias vector.

The training objective is the cross-entropy cost function, which is the negative log-likelihood of the true class label *y* ^(*k*)^ of each predicted sentence *S* ^(*k*)^:

9 $$ J\left(\theta \right)=-\frac{1}{l}\sum_{k=1}^l\log p\left({y}^{(k)}|{S}^{(k)}\right) $$

where *l* is the number of labelled sentences in the training set and the superscript *k* indicates the *k-*th labelled sentence. We applied RMSprop (Resilient Mean Square Propagation) to update parameters with respect to the cost function because RMSprop is empirically an appropriate optimization algorithm for learning the RNN-based model [41]. RMSprop does not need to adjust the learning rate manually in the process of iteration, and moreover, it has better convergence and fast convergence rate.

## Results and discussion

### Datasets

#### The training and test datasets

We trained the proposed model on the DDI-2013 corpus, including three datasets: DB-2013, ML-2013 and their union set (Overall-2013). All drug mentions and drug pairs in each sentence are annotated manually. Each drug pair is annotated as either no interaction or true interaction (the detection task), with more fine-gained annotations consisting of four labels: “*mechanism*”, “*effect*”, “*advice*” and “*int*” (the classification task). Table 1 lists the statistics of this corpus. A total of 330 and 62 negative instances on the DB-2013 and ML-2013 test datasets were filtered out by the basic preprocessing stage, respectively. In particular, for the ML-2013 dataset, we trained our system on the combination of the DB-2013 and ML-2013 training datasets, as suggested by [11].

**Table 1:** Statistics for DDI- 2013 corpus

Instances	DDI type	DB-2013		ML-2013	
		Training set	Test set	Training set	Test set
Positive	mechanism	1257	278	62	24
	effect	1535	298	152	62
	advice	818	214	8	7
	int	178	94	10	2
	Total	3788	884	232	95
Negative		22,217	4381	1555	401
Total		26,005	5265	1787	434

#### The pre-training corpus of embedding vectors

The pre-training corpus for word representations, which is approximately 2.5 gigabytes in size, consists of two parts. One part comes from all abstracts before 2016 that were obtained by querying the key word “drug” in the PubMed database, and the other one is the texts of the DDI-2013 corpus. Compared with the one-hot representation of POS tags, Zhao’s experiments [23] indicate that POS representations encoded by using an auto-encoder neural network model have a better effect on the performance of a system. However, vectors encoded by an auto-encoder might contain little semantic information. Therefore, our POS training corpus contains only sentences of the DDI-2013 corpus that are labelled with POS tags (43 POS tags). The two types of embedding vectors were trained by the *word2vec* tool (https://code.google.com/p/word2vec/) [42]. The position embedding vectors were initialized with random values that follow the standard normal distribution.

### Evaluation metrics

The performance of our system is evaluated by the standard evaluation measures (precision, recall and F-score). F-score is defined as *F* = (2*PR*) / (*P* + *R*), where *P* denotes the precision and *R* denotes the recall rate. F-score can play a balancing role between P and R.

### Hyperparameters

Keras library was used to implement our model. We tuned the hyperparameters of our model to optimize system performance by conducting 5-fold sentence-level cross-validation on the training set. The choices generated by this process are listed in Table 2.

**Table 2:** Hyperparameters

Parameter	Parameter name	Value
*m*_*1*_	Word emb.Size	200
*m*_*2*_ & *m*_*3*_	POS&position emb.Size	10
*n*	The length of a pruned sentence	85
Mini-batch	Minimal batch	128
LSTM dim.	the number of hidden units	230

**Fig. 2:**
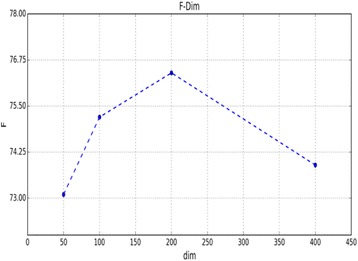
Evaluation of the dimensionality of word embedding when our model without the attention mechanism was trained

**Fig. 3:**
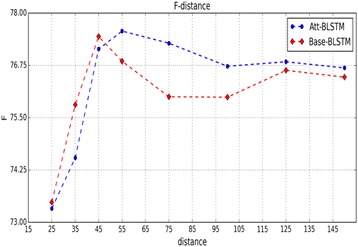
F-scores of the proposed model as the distance between candidate drugs becomes longer

The dimensionality of word-embedding vectors (*m*_*1*_ = 200 in our model) could affect system performance, as shown in Fig. 2. On the one hand, vectors do not contain enough semantic information because of the small dimension. On the other hand, with the increase in the dimension, embedding vectors bring much more noise despite their richer semantics. Meanwhile, a system needs to spend more training time with the increasing complexity of the model. The dimensionality *m*_*2*_ of the position-embedding vectors was set as 10, as used by Zeng [34]. Figure 3 shows that our model achieves the best F-score when the maximal separation between candidate drugs is less than 75, which contains all positive instances of three datasets. According to this result, we kept at least five extra words before and after two candidate drugs. Therefore, we set the length *n* of a pruned sentence as 85. We output results at each epoch. When the epoch numbers are nearly 131, 128 and 58 for the Overall-2013, DB-2013 and ML-2013 datasets, respectively, our model achieved better performance on the corresponding dataset. The number of hidden units (230) of LSTM was set as the same size of input dimension of the LSTM layer to simplify our research.

For RMSprop optimization, we set the learning rate *lr* = 0.001 and the momentum item parameter *rho* = 0.9, as suggested by Tieleman et al. [41]. To alleviate the over-fitting problem, dropout [43] was applied to the LSTM and softmax layers on feed-forward networks. Dropout has improved the performance of many systems because it reduces the interdependency of neural units by randomly omitting feature detectors from the network during the training. The dropout rate was set to 0.5 in our model, as used by Hinton et al. [43].

### Effects of input attention

**Fig. 4:**
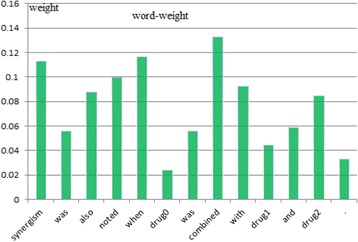
Input attention visualization. *Note*: Drug0 and drug1 are the candidate drug pair

We examined different score functions as described in section 2. The results in Table 3 show that candidate-drug-oriented score functions are superior in performance to those without the special target (e.g., *tanh*). The *tanh-score* function equivalently adds a hidden layer to the network, so performance drastically decreases with increasing complexity and noise of the model. Figure 4 shows an example for the word-level attention values calculated by *dot*-*score* used in our model, *Att-BLSTM*. We find that the words “*synergism*”, “*combined*” and “*when*” have higher attention values than other words. This seems sensible in light of the ground-truth labelling as an “*effect*” relationship (between *drug0* and *drug1*). Hence, we might infer that the introduction of input attention highlights those influential words and makes semantic relationships between candidate drugs clear. It can be seen from Table 5 that the F-score of the model with input attention increases 23.7% over that of the model without input attention when only word embedding is considered. On the one hand, this result supports the above conclusion to some extent. On the other hand, it also indicates that a high F-score can be achieved by only using word embedding for the proposed model. To analyse the effect of input attention on the distance between candidate drugs, we group sentences in which the distance is lower than a fixed length in the training and test datasets. Figure 3 shows that the performance of *Att-BLSTM* with input attention is distinctly superior to those of *Base-BLSTM* without an attention mechanism when the distance is greater than 50, whereas the *Base-BLSTM* is slightly better when the distance is less than 50. The following reasons may explain this phenomenon. For short sentences, the *Base-BLSTM* model might have the ability to learn adequately from its network. Input attention equivalently appends additional restrictions to the model and requires semantic meanings to match strictly, which causes *Att-BLSTM* to misclassify some sentences with ambiguous semantics. However, the bias characteristic of BLSTM causes some important information to be disregarded when a sentence becomes longer. Hence, *Base-BLSTM* misclassifies more negative instances as positive instances as the number of positive instances increases. However, input attention in the proposed model makes up for this shortcoming. *Att-BLSTM* misclassifies fewer false-positive instances (fp) than *Base-LSTM*, despite the fact that the number of true-positive instances (tp) recognized by *Att-BLSTM* has slightly decreased. Table 4 lists the number of different instances detected by *Base-BLSTM* and *Att-BLSTM* on the Overall-2013 dataset. Comparing tp and fp between *Base-LSTM* and *Att-BLSTM*, the decrease in fp for *Att-LSTM* is nearly three times that of tp (65 vs. 23). Therefore, it can be seen from Table 3 that the introduction of the attention layer increases the precision and decreases the recall for the two candidate-drug-oriented attention models.

**Table 3:** Performance of different score functions for the DDI classificaton on the Overall-2013 dataset

Score function	P(%)	R(%)	F(%)
Base_BLSTM	74.0	78.6	76.2
dot-score	78.4	76.2	77.3
cos-score	76.3	76.5	76.4
Tanh-score	67.9	65.9	66.9

Base_BLSTM is the BLSTM model without an attention mechanism which uses our all preprocessing techniques and all input embeddings including word, POS and position embedding

**Table 4:** The number of different instances detected by two models for the DDI classificaton on the Overall-2013 dataset

Model	tp	fp	fn	tp + fn
Base_BLSTM	769	270	210	979
Att-BLSTM	746	205	233	979

*tp* denotes the number of true-positive instances, *fp* denotes the number of false-positive instances, and *fn* denotes the number of false-negative instances

Nonetheless, input attention will not work when the distance between two drugs exceeds a threshold value. However, in practice, the distances used in our experiments should meet most of needs of relation classification at the sentence level.

### Effects of input representations

We conducted experiments to evaluate the effectiveness of the strategies adopted in our method. In addition to the three embedding vectors, our model also processes the following-based cataphora and prunes sentences. Tables 5 and 6 show the effects of these steps on the performance of our model.

**Table 5:** Performance changes with different input representations on the overall-2013 dataset

Input representation	P(%)	R(%)	F(%)
(1): word without attention	54.7	42.8	48.0
(2): word + att	76.5	67.5	71.7
(3): word + att + pos	70.9	74.7	72.7
(4): word + att + position	79.1	73.9	76.4
(5): word + att + pos + position	78.4	76.2	77.3

Every model in this table uses all preprocessing techniques of our approach. Word without attention denotes the model without the attention mechanism which uses only word embedding. Word + att denotes the model which uses the attention mechanism and word embedding

**Table 6:** Performance changes with different preprocessing procedures on the overall-2013 dataset

Processing procedure	P(%)	R(%)	F(%)
(1): only candidate drugs replaced	75.9	68.7	71.5
(2): basic processing	77.5	72.3	74.8
(3): (2) + Following anaphora	76.9	76.5	76.7
(4): (3) + Pruned Sentences	78.4	76.2	77.3

Every model in this table uses three input embeddings of our approach

The position feature is an important factor that influences performance. The F-score increases by 4.7% when position embedding is introduced. Our model with the position feature further intensifies the significant contextual combination by distinguishing semantic meanings from the current word and drug entities. Moreover, the proposed model further improves the F-score when POS embedding is incorporated. Furthermore, it can be seen from Table 6 that some preprocessing of the given sentences effectively improves the performance of our system. However, our model also performs well even if texts are not pre-processed.

### Performance comparisons with other systems

To evaluate our approach, we compared our system with top-ranking systems in the DDI tasks.

#### Other systems for comparison

For the DDI tasks, most existing systems are based on either SVM or NN. We compare the performance of the proposed model with those of the following baseline methods. All compared systems have performs well and have their own pros and cons.

1. **SVM-based methods:** SVM-based methods commonly depend either on carefully handcrafted features or on elaborately designed kernels, which replace the dot product with a similarity function between two vectors. RAIHANI [17] designs many rules and features such as chunk, trigger words, filtering negative sentence and SAME_BLOK. This system still designs different features for the SVM classifier of each subtype. FBK-irst [11] combines different characteristics of three kernels. UTurku [14] uses informatics from domain resources such as DrugBank, in addition to sentence and dependency features.
2. **NN-based methods:** NN-based methods learn the high-level representation of a sentence by the CNN or LSTM architecture. For CNN-based systems, MCCNN [21] uses multichannel word embedding vectors and SCNN [23] combines traditional features and embedding-based convolutional features. For LSTM-based methods, *joint AB-LSTM* combines two LSTM networks, one of which exploits the pooling attention. For the above methods, word embedding is an indispensable feature; position embedding is used by all of them except MCCNN, and some filtering techniques are exploited to rule out irrelevant negative instances, in addition to common preprocessing techniques for texts.

#### Overall performance

Table 7 shows that our model achieves the best performance on the Overall-2013 test dataset for both DDI detection (*DEC*) and DDI classification (*CLA*). Our F-scores for the two tasks are 2.2% and 5.8% higher than those of current best results, respectively. In addition, we observe from Table 8 that our *Att-BLSTM* has the characteristics of both higher precision and higher recall on the three datasets, while most existing systems have relatively lower recall values. To give every model a fair comparison, Table 9 lists performance of NN-based systems on the overall-2013 dataset for DDI classification if systems don’t use main text processing techniques. Our model only replaces the candidate drugs (row (1) in Table 6), while other systems use basic text processing and replaced candidate drugs (negative instances aren’t filtered). We don’t provide comparisons with SVM-based systems. One of the reasons is that these systems have the different kind of architecture with ours. Moreover, most systems didn’t provide their source codes, and their papers didn’t present results of text preprocessing on the overall-2013 dataset either.

**Table 7:** Performance comparisons (F-score) with top-ranking systems on the overall-2013 dataset for DDI detection and DDI classification

Method	Team	CLA	DEC	MEC	EFF	ADV	INT
SVM	RAIHANI [17]	**71.1**	81.5	**73.6**	69.6	**77.4**	**52.4**
	Context-Vector [15]	68.4	**81.8**	66.9	**71.3**	71.4	51.6
	Kim [16]	67.0	77.5	69.3	66.2	72.5	48.3
	FBK-irst [11]	65.1	80.0	67.9	62.8	69.2	54.7
	WBI [12]	60.9	75.9	61.8	61.0	63.2	51.0
	UTurku [14]	59.4	69.9	58.2	60.0	63.0	50.7
NN	*joint AB-LSTM* [32]	**71.5**	**80.3**	**76.3**	67.6	**79.4**	43.1
	MCCNN [21]	70.2	79.0	72.2	68.2	78.2	**51.0**
	Liu CNN [22]	69.8	–	70.2	**69.3**	77.8	48.4
	Zhao SCNN [23]	68.6	77.2	–	**–**	–	–
Ours	Att-BLSTM	**77.3**	**84.0**	**77.5**	**76.6**	**85.1**	**57.7**

The listed results come from the corresponding papers. The symbol “-” denotes no corresponding values, because the related paper did not provide complete results (similarly hereinafter). “*DEC*” only indicates DDI **detection**. “*CLA*” indicates DDI **classification**. “*MEC*”, “*EFF*”, “*ADV*” and “*INT*” denote “*mechanism*”, “*effect*”, “*advice*” and “*int*” types, respectively. The highest scores are highlighted in bold

**Table 8:** Performance comparisons (F-score) with top-ranking systems on the three datasets

Method	Team	DB-2013			ML-2013			Overall-2013		
		P(%)	R(%)	F(%)	P(%)	R(%)	F(%)	P(%)	R(%)	F(%)
SVM	RAIHANI [17]	–	–	**74.0**	–	–	43.0	**73.7**	**68.7**	**71.1**
	Context-Vector [15]	–	–	72.4	–	–	**52.0**	–	–	68.4
	Kim [16]	–	–	69.8	–	–	38.2	–	–	67.0
	FBK-irst [11]	66.7	**68.6**	67.6	41.9	37.9	39.8	64.6	65.6	65.1
	WBI [12]	65.7	60.9	63.2	45.3	**30.5**	36.5	64.2	57.9	60.9
	UTurku [14]	**73.8**	53.5	62.0	**59.3**	16.8	26.2	73.2	49.9	59.4
NN	MCCNN [21]	–	–	70.8	–	–	–	**76.0**	**65.3**	**70.2**
	Liu CNN [22]	**77.0**	66.7	**71.5**	**61.4**	**45.3**	**52.1**	75.7	64.7	69.8
	Zhao SCNN [23]	73.6	**67.0**	70.2	39.4	39.1	39.2	72.5	65.1	68.6
Ours	Att-BLSTM	**78.9**	**75.7**	**77.3**	**71.8**	**59.0**	**64.7**	**78.4**	**76.2**	**77.3**

The highest scores are highlighted in bold

**Table 9:** Performance comparisons (F-score) with NN-based systems on the overall-2013 dataset for DDI classification if systems don’t use main processing techniques

Method	Team	P(%)	R(%)	F(%)
NN-based	joint AB-LSTM [32]	71.3	66.9	69.3
	MCCNN [21]	–	–	67.8
	Liu CNN [22]	75.3	60.4	67.0
	Zhao SCNN [23]	68.5	61.0	64.5
Our model	Att-BLSTM	**75.9**	**68.7**	**71.5**

The listed results come from the corresponding papers. The symbol “-” denotes no corresponding values, because the related paper did not provide complete results. Our model only replaces the candidate drugs (row (1) in Table 6), while other systems use basic text processing and replaced candidate drugs (negative instances aren’t filtered). The highest scores are highlighted in bold

#### Performance on interaction types

In addition, as far as the performance of all four interaction types are concerned, Table 7 shows that *Att-BLSTM* far surpasses other systems. The four interaction types in descending order of the degree of classification difficulty are “*int*”, “*effect*”, “*mechanism*” and “*advice*”. The performance of each subtype is shown in Table 10. Compared with the CNN-based and other LSTM-based systems, *Att-BLSTM* achieves obvious increases in F-score of 6.7% and 14.6% on the “*int*” type with the fewest training and test instances and of 8.4% and 9.0% on the “*effect*” type with high semantic complexities, respectively. Moreover, our F-scores on the “*mechanism*” and “*advice*” types show more than 1.2% and 5.7% relative improvements compared with the best values, respectively.

**Table 10:** Performance of interaction types on the overall-2013 dataset

Subtype	P(%)	R(%)	F(%)
EFF	71.9	81.9	76.6
MEC	84.1	71.9	77.5
ADV	84.8	85.5	85.1
INT	75.0	46.9	57.7

#### Performance on the ML-2013 and DB-2013 datasets

To compare the performance on different types of documents, the results from the ML-2013 and DB-2013 datasets are shown in Table 8. It should be noted that our performance represents a significant improvement on the ML-2013 dataset for DDI classification. Our F-score exceeds those of the best existing systems by more than 12.6%. Meanwhile, our F-score on the DB-2013 dataset outperforms those of the best NN-based and SVM-based systems by more than 5.8% and 3.3%, respectively.

#### Performance analysis

The associated contexts of a word might have a longer span due to the characteristics of the DDI-2013 corpus. Therefore, it is especially important to capture relations among long-range words. However, some global and possibly non-consecutive patterns cannot be adequately learned by CNN-based and SVM-based systems. Although RAIHANI [17] has the best F-score of the SVM-based systems, this system depends too much on NLP toolkits and manual intervention. For CNN-based systems [21–23], Zhang’s experiments [24] demonstrate that CNN splits the semantic meaning into separate word segments and mixes them together so that it learns only local patterns for classification tasks.

By contrast, our approach has the ability to identify the dependency patterns of long-distance words. There are two main reasons for this. One reason is to exploit the characteristic of LSTM that adaptively accumulates contextual information word by word. The semantic meaning (the output at the last time step) of our BLSTM layer with fitted input features is actually based on contributions of all words in a sentence. Thus, our model can adequately capture the integrated contextual information. Experiments [24] indicate that RNN-based systems are similar to CNN-based systems in performance when the distance among interdependent words has a relatively small span; otherwise, RNN has clear advantages over CNN. In this respect, the *joint AB-LSTM* model [32] uses the same BLSTM as ours.

The other reason for the significant improvement in our performance may be the contribution of input attention that targets the candidate drugs. It provides more targeted semantic matching and causes our model to explicitly find important cue words. Consequently, the introduction of input attention further enhances the memory of LSTM of influential segments for classifying the relation between a long-distance drug pair. Therefore, our model is able to classify DDI types effectively (see Fig. 3). Finally, the above two reasons also lead to a large margin of performance improvement on the ML-2013 dataset with sentences of more complex subordinated structures. In the case of the attention mechanism, although the *joint AB-LSTM* model [32] also intends to exploit the attention mechanism to capture the important clues for DDI classification, their experiments demonstrate that the introduced attentive pooling degrades rather than increases their F-score. The reason for this finding may be that their introduced attentive pooling is a non-targeted general approach rather than a target-specific approach. Moreover, their system has higher time and model complexities compared with our system because of the combination of two LSTM models.

Furthermore, although the use of techniques to filter negative instances by most of the systems partly balances the biased dataset, many positive instances are removed from the training set, which causes their model to lose many learning opportunities.

## Conclusion

In this study, we applied a neural network model, *Att-BLSTM*, based on RNN with LSTM units and an attention mechanism for classifying DDIs from biomedical texts. By introducing input attention, our model overcomes the bias deficiency of LSTM to some extent, which omits some important previous information when processing long sentences. The proposed model only depends on three input embedding vectors and the simple network architecture. Our model achieves a good overall performance on the detection and classification tasks for the DDI-2013 corpus. In particular, our F-score far outperforms the current best F-score by 12.6% on the ML-2013 dataset, which indicates the effectiveness of our approach. Experimental analysis indicates that our approach can effectively recognize not only close-range but also long-range patterns among words in long and complex sentences.
